# InflamNat: web-based database and predictor of anti-inflammatory natural products

**DOI:** 10.1186/s13321-022-00608-5

**Published:** 2022-06-04

**Authors:** Ruihan Zhang, Shoupeng Ren, Qi Dai, Tianze Shen, Xiaoli Li, Jin Li, Weilie Xiao

**Affiliations:** Key Laboratory of Medicinal Chemistry for Natural Resource, Ministry of Education; Yunnan Provincial Center for Research & Development of Natural Products, School of Chemical Science and Technology, and School of Software, Yunnan University, Kunming, 650091 China

**Keywords:** Anti-inflammation, Natural products, Machine learning, Web platform

## Abstract

Natural products (NPs) are a valuable source for anti-inflammatory drug discovery. However, they are limited by the unpredictability of the structures and functions. Therefore, computational and data-driven pre-evaluation could enable more efficient NP-inspired drug development. Since NPs possess structural features that differ from synthetic compounds, models trained with synthetic compounds may not perform well with NPs. There is also an urgent demand for well-curated databases and user-friendly predictive tools. We presented a comprehensive online web platform (InflamNat, http://www.inflamnat.com/ or http://39.104.56.4/) for anti-inflammatory natural product research. InflamNat is a database containing the physicochemical properties, cellular anti-inflammatory bioactivities, and molecular targets of 1351 NPs that tested on their anti-inflammatory activities. InflamNat provides two machine learning-based predictive tools specifically designed for NPs that (a) predict the anti-inflammatory activity of NPs, and (b) predict the compound-target relationship for compounds and targets collected in the database but lacking existing relationship data. A novel multi-tokenization transformer model (MTT) was proposed as the sequential encoder for both predictive tools to obtain a high-quality representation of sequential data. The experimental results showed that the proposed predictive tools achieved an AUC value of 0.842 and 0.872 in the prediction of anti-inflammatory activity and compound-target interactions, respectively.

**Supplementary Information:**

The online version contains supplementary material available at 10.1186/s13321-022-00608-5.

## Introduction

Inflammation is the response of the immune system to pathogen infection and tissue injury caused by physical or chemical factors, and it is essential for the body’s defense against harmful stimuli. However, uncontrolled and prolonged inflammation can induce or contribute to the occurrence of many diseases, such as autoimmune disease, Alzheimer’s disease, cancer, diabetes, and others. Natural products (NPs) are an important source for drug discovery since they have unique molecular structures that differ from synthetic compounds, as well as possessing ideal pharmacokinetic features, with examples from historical aspirin (inspired by willow extracts) to recent Fingolimod (optimized from myriocin, for multiple sclerosis treatment) [1, 2]. As revealed in our previous cheminformatics study, anti-inflammatory NPs occupy a large and diverse structural space, with flavonoids and triterpenoids being the major types. Although hundreds of NPs with cell-based anti-inflammatory activity have been reported, more than 2/3 of them have no known targets [3]. In-depth research of NP-inspired drug leads was limited by unmanageable factors during NPs discovery, such as structure, quantity, and function. Computational and informatics tools have been applied in many aspects of the early phase of drug discovery, such as target identification, hits screening, and lead optimization [4]. There are tens of NP-specific databases currently available in bioactive NP research, and several studies have reported the use of machine learning algorithms to predict the bioactivity or targets of NPs [5].

The applicability domain of a machine learning-based bioactivity prediction model is crucial for its performance. Since NPs possess different structural features from synthetic compounds, models trained with synthetic compounds may not perform well with NPs [6]. Therefore, there is still a demand for well-curated databases and user-friendly predictive tools that are optimized for NP-inspired drug discovery.

In this study, we presented the development of a comprehensive web-based platform for anti-inflammatory NP research that combines a database and predictive tools. First, the InflamNat website offered an easy way of accessing anti-inflammatory NP information, including their structure, physicochemical properties, cell-based anti-inflammatory activities, and identified molecular targets.

Furthermore, the anti-inflammatory effect of customized NPs and undiscovered targets of the compounds could be predicted using the InflamNat database. Two machine learning-based predictive tools were specifically designed for natural products that (a) predict the anti-inflammatory activity of natural products and (b) predict the compound-target relationship for the compounds and targets collected in the database but lacking existing relationship data. InflamNat used sequence data, such as the Simplified Molecular Input Line Entry System (SMILES) of drugs and the amino acid sequences of proteins, as inputs to train machine learning-based predictive tools. Notably, a novel sequence representation model Multi-Tokenization Transformer model (MTT) was the proposed feature encoder of ML-based predictive tools to produce comprehensive and high-quality sequence representation of the compounds (SMILES) and proteins (amino acid sequences). Most existing sequence representation models [12] employ a tokenized method to obtain tokens. In comparison, MTT improves the quality of contextualized representation of sequence data using multiple tokenizations of sequence data. Our anti-inflammatory NP experimental results demonstrated that the proposed tools achieved the desired performance.

## Methods

### Data collection

The InflamNat database was initially composed of 665 compounds and has since been increased to 1351 compounds [3]. The structures of NPs and cellular anti-inflammatory activities were collected from 319 research articles published between 2000 and 2020. The structures were stored as SMILES, canonical SMILES, and InChiKey. The names of the compounds were recorded as they were in the references, and other synonyms were acquired from PubChem and ChEMBL, if applicable [7, 8]. The following criteria were used for the selection of cell-based anti-inflammatory bioassay data: (1) the assays were performed in inflammatory cell models (e.g. macrophages); (2) the collected data included not only the production of inflammatory factors (nitric oxide (NO), PGE2) and cell cytokines (IL-1β, IL-6, IL-12, TNFα) but also the cytotoxicity to exclude the effects of cell viability. The origin of the NPs represented the organism (genus and species) that produced the compounds in the cited reference, and “WD” stood for “widely distributed”. The targets of InflamNat compounds were collected from ChEMBL, which were then filtered to keep only the “single protein” type of targets, ensuring that the data reflected the direct compound-target interaction.

For the construction of training datasets, the compounds were classified as ACTIVE or INACTIVE based on their inhibition of NO production, and compound-target interaction, using unified criteria: ACTIVE was defined as IC_50_/EC_50_ < 50 μM, whereas INACTIVE was defined as IC_50_/EC_50_ > 50 μM.

The complete datasets can be downloaded from the home page of the website (Additional files 1, 2, 3, 4). In order to avoid being misled, only the ACTIVE targets of the compounds are shown in the online database.

### Cheminformatic analysis

The general properties of InflamNat compounds, including molecular weight (MW), Log (ALogP), topological polar surface area (TPSA), the number of hydrogen bond donors (#HBD), the number of hydrogen bond acceptors (#HBA), and the number of rotatable bonds (#RotB) were determined using RDKit [9]. The Bemis-Murcko scaffolds were extracted using the ChemMine package with the R program [10], with only the largest scaffold retained for each compound. Principle component analysis (PCA) was performed to investigate the chemical space of the InflamNat compounds and around 4000 approved drugs collected in DrugBank [11]. Molecular features that were used for PCA, including MW, LogP, TPSA, #HBD, #HBA, and #RotB, were determined using RDKit.

### Multi-tokenization transformer model

The proposed predictive tools in InflamNat used sequential data, such as Simplified Molecular Input Line Entry System (SMILES) of drugs and the sequence of amino acids of proteins to train the ML-based prediction model. Our proposed predictive tools were trained in an end-to-end learning manner. Therefore, the issue of feature representations of the molecule and protein needed to be addressed before the development of the prediction model.

Although various NLP-inspired representation models for molecules and proteins, such as [12, 13], have been proposed in recent years to address problems in many pharmaceutical and life science applications, the tokens, which are the basic unit in sequential models, are typically pre-specified using a single tokenizer. For example, they have considered compound substructures derived from the Morgan algorithm for molecules [14], and individual AAs for proteins [15]. Notably, there is no standard tokenizer for molecules or proteins. Various tokenizers can provide different lexical component units with different semantics. Therefore, it was logical to investigate whether a sequence representation model integrating multiple tokenization could provide a more comprehensive and high-quality sequence representation.

**Fig. 1:**
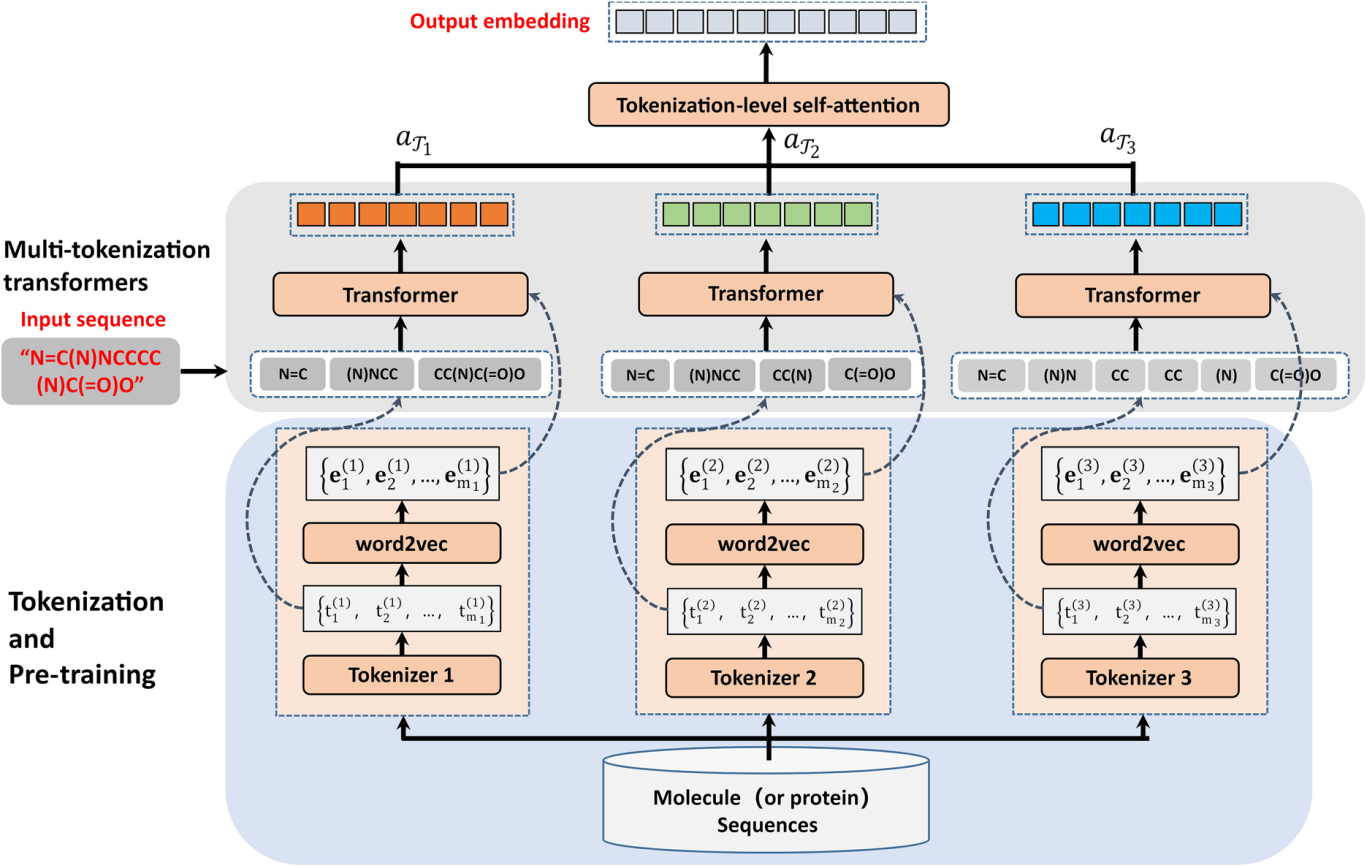
The framework of the multi-tokenization transformer model (MTT) which employs various sequence tokenized approaches and multiple transformers to obtain a high-quality representation of sequential data. MTT is composed of three different modules: multi-tokenization and pre-training, multiple transformers-based encoder, and tokenization-level self-attention. As a feature encoder, MTT combines the downstream prediction model into a unified end-to-end neural network learning framework

In this study, we presented a novel sequence representation learning model, the Multi-Tokenization Transformer model (MTT), which employs various sequence tokenized approaches and multiple transformers [18] to obtain a high-quality representation of sequential data. Figure 1 displays an overview of MTT. Overall, MTT consisted of three modules: multi-tokenization and pre-training, multi-transformers-based encoder, and tokenization-level self-attention.

#### Multi-tokenization and pre-training module

Given the drug molecule (or protein) sequence corpus, various token sets of the corpus were obtained through different tokenizers. Each token set was considered as a set of words, then the Word2vec algorithm [17] was used for self-supervised pre-training to obtain the token embeddings.

#### Multi-transformers-based encoder module

When a new sequence data $s$ was input, different tokenizers will divide $s$ into tokens. For example, for the input SMILES “N=C(N)NCCCC(N)C(=O)O” of the drug, various tokenizers deal with the SMILES and produce the 1st tokenization “N=C|(N)NCC|CC(N)C(=O)O”, the 2nd tokenization “N=C|(N)NCC|CC(N)|C(=O)O”, and the 3rd tokenization “N=C|(N)N|CC|CC|(N)|C(=O)O” (Different tokens are separated by the symbol | in tokenization). In specific tokenization, the initial feature representation of the tokens was obtained through pre-training embedding of the tokenization. These initial token embeddings were then input into a tokenization-specific transformer encoder to yield contextualized representations through its layers.

#### Tokenization-level self-attention module

Because different tokenization may contribute differently to the final predicator, a self-attentive weight was introduced to determine tokenization importance. In particular, we used a self-attentive mechanism [18] to calculate the importance of each tokenization and output the final contextualized representation. Specifically, the self-attentive weight was calculated using:

1 $${a}_{\mathcal{T}}=\frac{\mathrm{exp}\left(\mathrm{MLP}\left({\mathbf{h}}_{\mathcal{T}}\right)\right)}{{\sum }_{{\mathcal{T}}^{\mathrm{^{\prime}}}}\mathrm{exp}\left(\mathrm{MLP}\left({\mathbf{h}}_{{\mathcal{T}}^{\mathrm{^{\prime}}}}\right)\right)}$$

where ${\mathbf{h}}_{{\mathcal{T}}^{^{\prime}}}$ denotes the contextualized embedding from a tokenization-specific transformer. Finally, the final contextualized representation is obtained by

2 $$\mathbf{h}={\sum }_{\mathcal{T}}{a}_{\mathcal{T}}{\mathbf{h}}_{\mathcal{T}}$$

It is worth noting that tokenization-level self-attention provided an explanation for sequential representation because self-attentive weight can be used to indicate the importance of different tokenization.

InflamNat used MTT as a feature encoder to provide contextualized representations of drug SMILES and protein sequences.

### Prediction models

InflamNat provides two machine learning-based predictive tools specifically designed for natural products that (a) predict the anti-inflammatory activity of natural products (AI-A) and (b) predict the compound–target relationship (C–T) for compounds and targets collected in the database but lacking existing relationship data.

AI-A is used to evaluate the anti-inflammatory potential of a natural product and is considered a binary classification that predicts whether a natural product compound has anti-inflammatory activity or not. The AI-A model uses the SMILES sequence of compound molecules as the input and MTT as the encoder to obtain the feature representation of compound molecules. An MLP is then used as the prediction model to yield the prediction result. Cross-entropy was used as the loss function for training the model.

C–T can predict the relationship between compounds and targets that were collected in the database without experimentally verified data. Since the molecular targets of many anti-inflammatory natural products have yet to be identified, this tool is useful for in-depth study and repurposing of these compounds. The C–T model uses both the SMILES sequence of compound molecules and the target protein sequence as inputs. In addition, both SMILES and protein sequences are tokenized by various tokenizers. MTT is then employed as an encoder to obtain the feature representations of compounds and proteins. The compound and protein representations are concatenated into a new feature vector, which is then input into an MLP classifier for prediction. Cross-entropy was used as the loss function of the model.

## Results and discussion

### Chemical space of InflamNat database

**Fig. 2:**
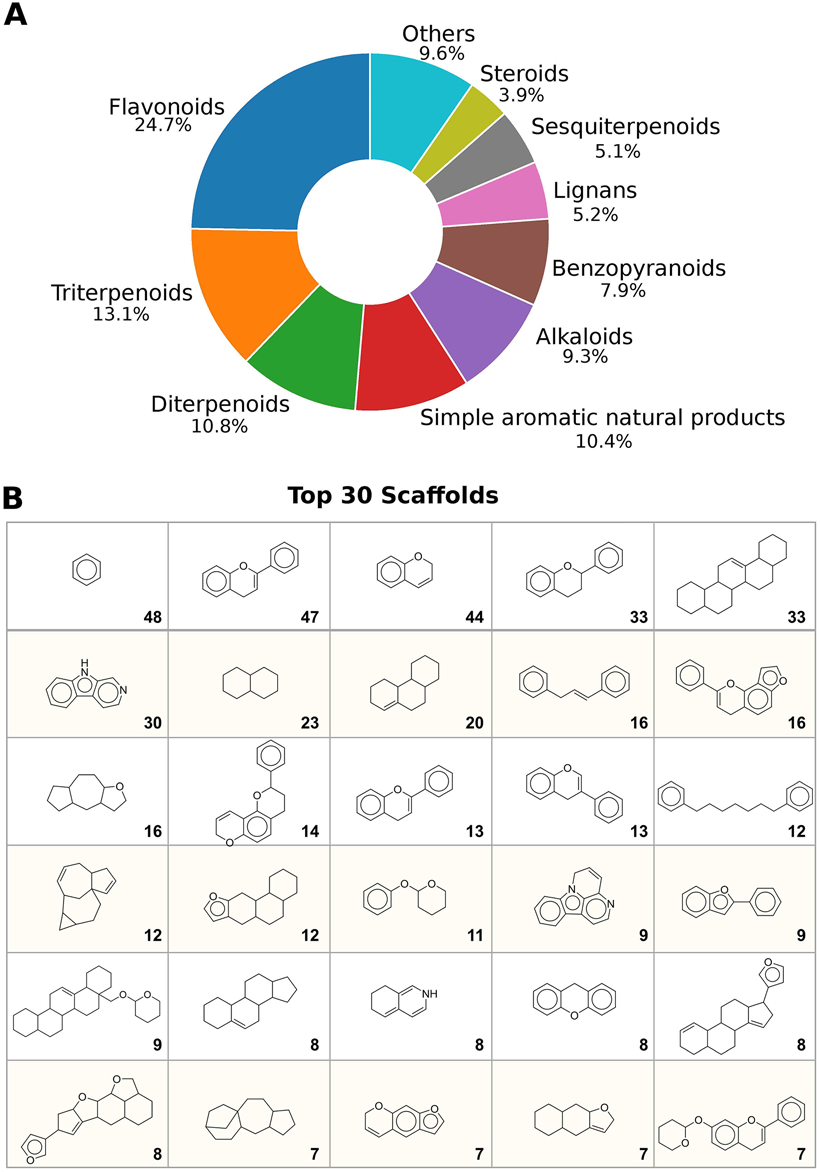
An overview of the structures in the InflamNat database. **A** The distribution of natural product structure classes. **B** The top 30 Bemis-Murcko scaffolds in the database. The number of compounds containing the scaffold are labeled in the bottom-right corner

Among the 1351 InflamNat compounds, the largest structure class is flavonoid, followed by triterpenoid, and diterpenoid (Fig. 2A). As discussed in our previous study, these structural classes are most frequently acquired and reported in the isolation of natural products. Furthermore, the phenolic hydroxyl groups and aromatic rings in flavonoids may contribute to their wide range of bioactivities by forming intermolecular interactions with protein targets. Triterpenoids possess a similar structure to steroid hormones, which play important roles in modulating immunological reactions [3]. The scaffolds of the NPs identified in InflamNat are very diverse (Fig. 2B), ranging from simple aromatic natural products with a single ring to complicated skeletons with a 5–6 ring system.

**Fig. 3:**
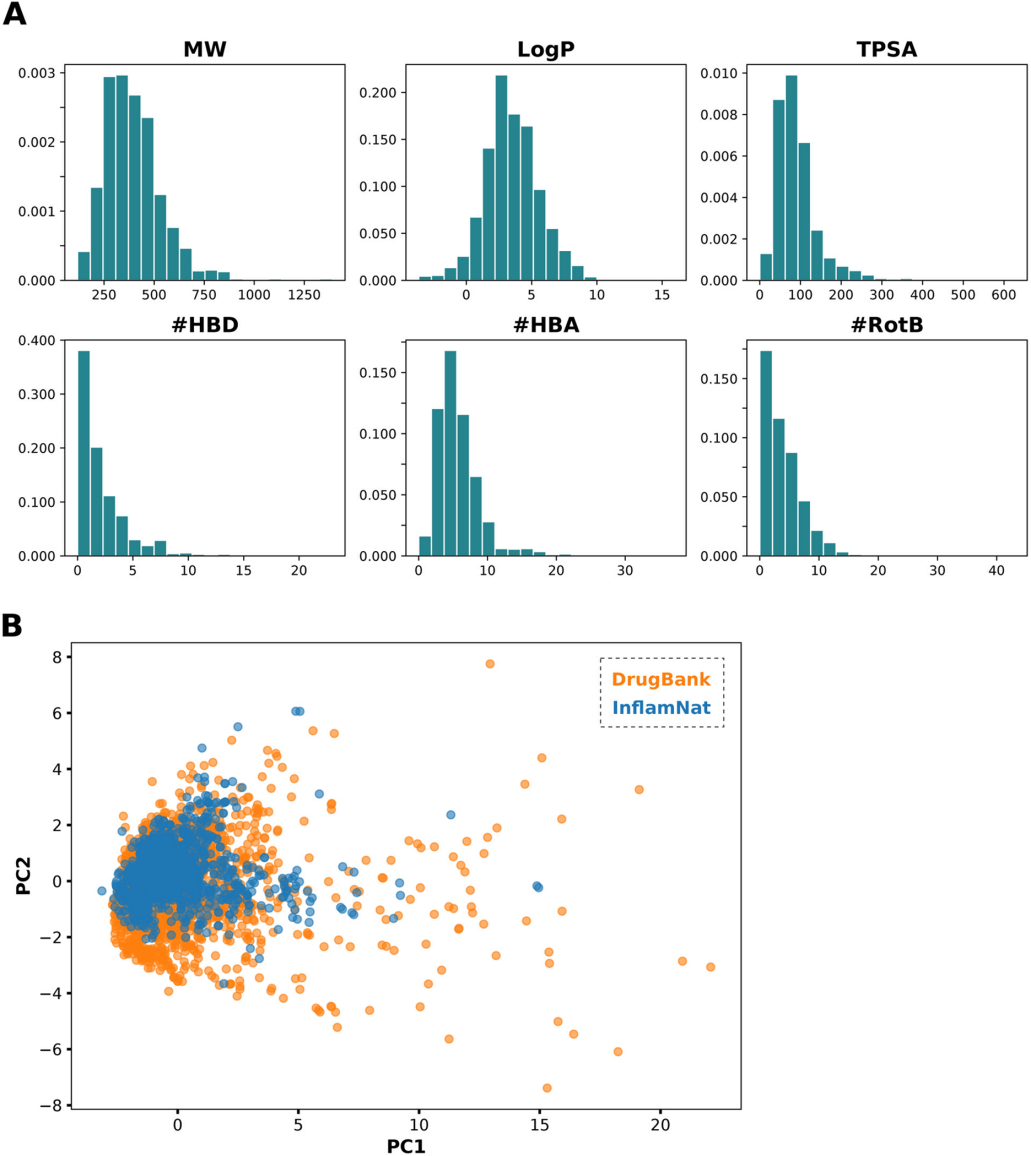
The (**A**) physicochemical properties and (**B**) chemical space of InflamNat compounds. MW: molecular weight. TPSA: topological polar sur-face area. #HBD: number of hydrogen bond donors, #HBA: number of hydrogen bond acceptors, #RotB: number of rotatable bonds

The distribution of physicochemical properties of InflamNat compounds is shown in Fig. 3A. According to Lipinski’s rule, 60% of the InflamNat compounds are drug-like (MW < 500, LogP < 5, #HBD < 5, #HBA < 10 and#RotB < 10), while 29% have a topological polar surface area (TPSA) < 60, indicating their potential to cross the blood–brain barrier (BBB). As shown in Fig. 3B, InflamNat compounds cover a similar but smaller chemical space compared to approved drugs.

### Bioactivity overview of InflamNat compounds

**Fig. 4:**
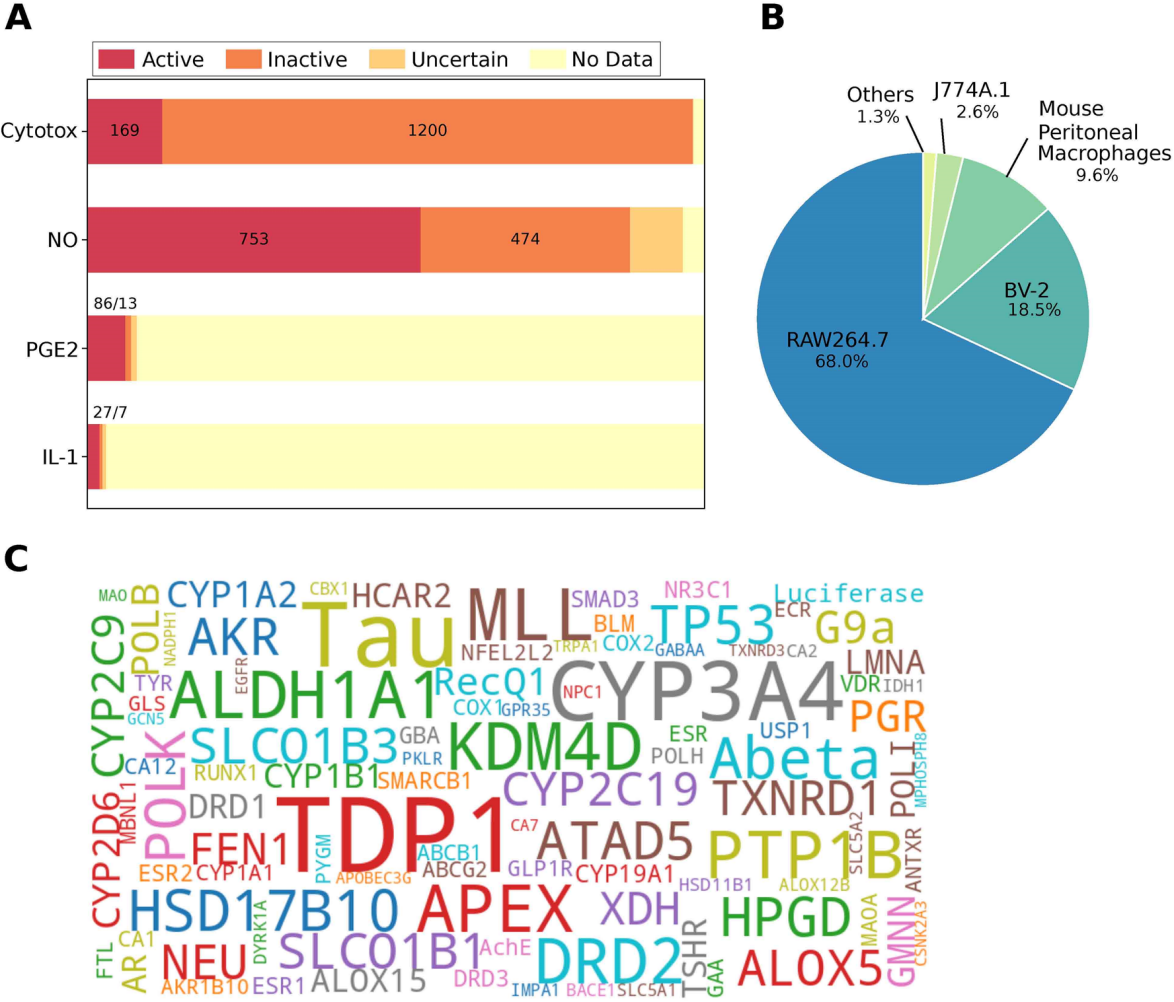
An overview of the bioactivities recorded in the database. **A** The major cellular anti-inflammation indices: cytotoxicity, and inhibition of NO, PGE2, and IL-1 production. The number of active and inactive compounds are labeled. **B** The cell types in which the cellular anti-inflammatory activities were measured. **C** Top 100 molecular targets of InflamNat compounds. The length of the target name corresponds to the number of records

The anti-inflammatory activity of InflamNat compounds in cells was obtained from the literature. In addition to the major indices, such as the inhibitory effect on the production of NO, PGE2, IL-1, IL-6, IL-8, and TNFα, cytotoxicity data were collected to exclude the effects of cell viability on the production of inflammatory factors. It was discovered that the inhibition of NO production was the most frequently reported data. Notably, NO production only represented specific inflammation signaling pathways, such as the classical NF-κB pathway, whereas other pathways may have different indices, such as IL-1β. However, data on the inhibition of the production of IL-1β and other inflammatory factors were insufficient to develop a machine learning model (Fig. 4A). Therefore, only the inhibitory activity of NO production was selected to train the prediction model of anti-inflammatory activity.

Since the anti-inflammatory effects were sensitive to the cellular model, the cell types used in the assays were also recorded (Fig. 4B), with the majority of the assays performed in mouse macrophage models (including RAW264.7 and J774A.1). The mouse microglial cell line BV-2 are macrophages residing in the central nervous system. The data acquired in macrophages were selected for model construction.

Only about 1/3 of InflamNat compounds were protein targets. The top 100 targets of InflamNat are listed in Fig. 4C. The length of the protein names corresponded to the frequency with which the protein appeared in the records. The targets of InflamNat compounds were related to a wide range of diseases, including cancer (Tyrosyl-DNA Phosphodiesterase 1, TDP1), anti-inflammation (15-Hydroxy-prostaglandin dehydrogenase, HPGD), nervous system disease (Amyloid-β, Abeta), and diabetes (Protein Tyrosine Phosphatase 1B, PTP1B). Enzymes related to drug metabolism, such as the cytochrome P450 proteins (CYPs), represented another type of target.

### Model training and prediction performance evaluation

The machine learning-based predictive tools in InflamNat, namely AI-A and C-T, were implemented based on the open-source machine learning framework Pytorch (https://pytorch.org). The details of model training and evaluated results for AI-A and C–T are presented in this subsection. Ten-fold cross-validation was used for experimental evaluation, in which experimental datasets were divided into ten parts. One part was used as the test dataset, another was used as the validation dataset, and the remaining eight parts were used as the training set. First, the training and verification sets were used for training and verification, and the test set was used for testing. The dataset of each part was used as a test set in turn, and the average classification accuracy obtained by ten-fold cross-validation was used to evaluate the performance of the classifier. In these experiments, all compounds adopted canonical SMILES sequence. In this study, the receiver operating characteristic curve (ROC curve for short) and the AUC value of the area under the curve were used to evaluate the prediction performance of the proposed model. All experimental tests were carried on a Windows 10 operating system with a Dell Precision T5820 workstation computer with an intel W-2145 8 core, 3.7 GHz CPU, and 64 G memory.

#### Tokenization and pre-training

A total of 1,938,745 canonical SMILES sequences were collected from ChEMBL [8], and 476,715 protein sequences from UniProt [19] as a corpus for pre-training. For SMILES compounds, Byte pair encoding (BPE) [20] and Extended-Connectivity Fingerprints (ECFP) [14] were used to produce tokens. BPE is a data-driven tokenization algorithm that is described in detail in [21]. BPE first learns a vocabulary of high-frequency SMILES substructure from a large chemical dataset (ChEMBL was used in this study), then tokenizes SMILES based on the learned vocabulary for the actual training of deep learning models. ECFPs are a type of fingerprint method that is specifically designed to capture molecular characteristics associated with the molecular activity. In ECFP, all substructures surrounding all heavy atoms of a molecule within a defined radius are generated and assigned unique identifiers. In our study, radii of 1 and 2 were used, thus they were called ECFP1 and ECFP2, respectively.

**Fig. 5:**
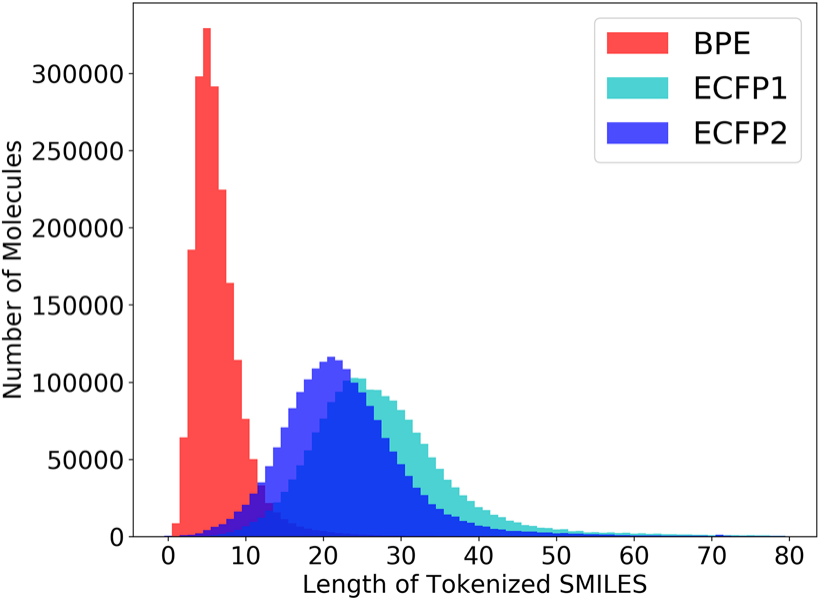
Distribution of number of tokens in various tokenized canonical SMILES of ChEMBL. The horizontal coordinate represents the number of tokens contained in a drug molecule, and the vertical coordinate represents the number of drug molecules containing a specific number of tokens

Figure 5 displays the statistical results of BPE, ECFP1, and ECFP2 tokenization for the collected ChEMBL dataset. The mean lengths for BPE, ECFP1, and ECFP2 tokenization were approximately 6, 22, and 25 tokens, respectively. According to the results, different tokenization methods provided different token sets, which resulted in different sequence partition semantics. For protein sequences, k-mers [22] and BPE were adopted to generate various tokens.

The tokens were considered as “words” and compounds (or proteins) as “sentences”. The Word2vec algorithm was then applied to the drug (or protein) corpus to obtain high-dimensional embeddings of tokens, where the vectors for chemically related tokens occupied the same part of vector space. These token embeddings were used as the initial feature representation of drugs (or proteins).

#### Training and evaluation of AI-A

According to the experimental requirements of a ten-fold cross-division, 890 NPs compounds molecular labeled by anti-inflammatory activity (represented by 1) and inactivity (represented by 0) were used to train the MTT-based encoder and binary classifier.

**Fig. 6:**
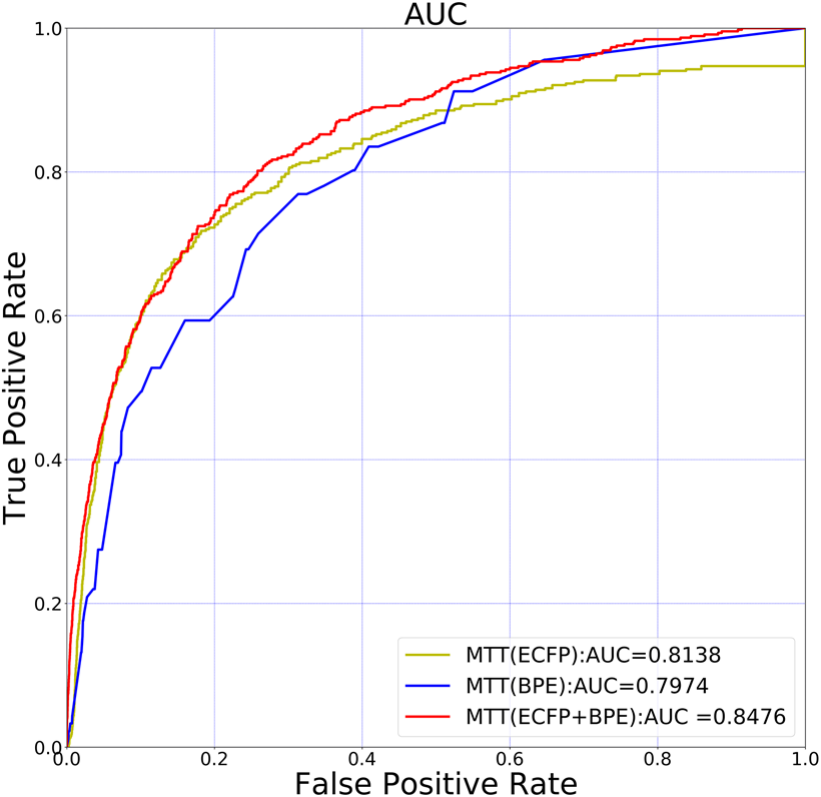
A prediction performance comparison of various classifiers using different tokenization. MTT(ECFP) represents the classifier using MTT with ECFP1 and ECFP2 tokenization. MTT(ECFP + BPE) represents the classifier using MTT with ECFP1, ECFP2, and BPE tokenization. Exper-imental results show that the adoption of multiple tokenization can improve prediction performance

After fine adjustment of model parameters, the dimension of the feature vector was set at 128, the heads of attention of the transformer at 6, the layer number of transformers at 5, and the learning rate at 0.01. Figure 6 shows the prediction performance comparison between MTT(ECFP) (MTT(ECFP) represents the classifier using the MTT encoder and ECFP represents tokenization), MTT(BPE), and MTT(ECFP + BPE). The results revealed that the adoption of multiple tokenizations can improve prediction performance. Finally, MTT with AUC 0.8476 was obtained.

**Fig. 7:**
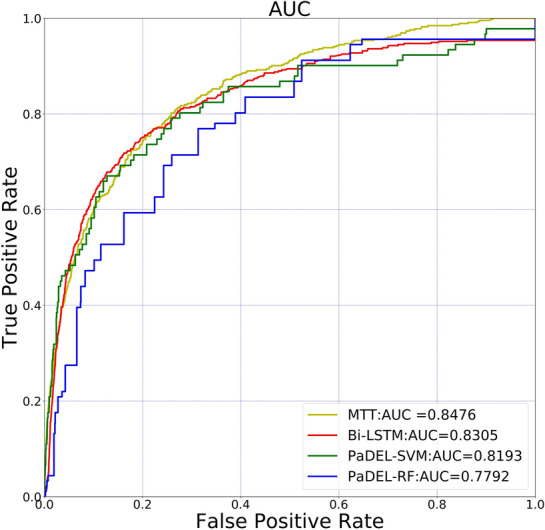
A prediction performance comparison of our proposed meth-od with other predictors. The experimental results show that the MTT-based predictor is superior to other baseline methods in terms of AUC

In order to evaluate the effectiveness of MTT with multi-tokenization, we compared the prediction performance of MTT-based classifier with other methods in our NPs classification datasets, such as SA-BiLSTM [12], PaDEL-SVM, PaDEL-RF. PaDEL-SVM and PaDEL-RF represented prediction methods using PaDEL [23] for compound description whereas and SVM and random forest as classifier, respectively. The comparison is shown in Fig. 7.

#### Training and evaluation of C–T

The aim of C–T was to predict the interactions between the compounds and targets. In this study, C–T was still modeled as a binary classification problem to classify the given compound-protein pair interaction or not. MTT was used as the encoder for both compound SMILES and protein sequences. After obtaining the embedding of the compound-protein pair, the embedding was input into the MLP-based classifier, which produced the final interaction score.

A total of 9126 compound-protein pairs labeled “1” (means compound-protein interact) or “0” (not interact) were used as datasets for the training prediction model. The datasets included 325 compounds and 796 proteins, with 7164 positive pairs (“1”) and 1962 negative pairs (“0”).

Ten-fold cross-validation was used to evaluate the prediction performance of the C–T model. Specifically, 10% of both the positive and negative pairs were randomly selected from the positive and negative datasets as the test set. The remaining pairs were used as training sets.

**Fig. 8:**
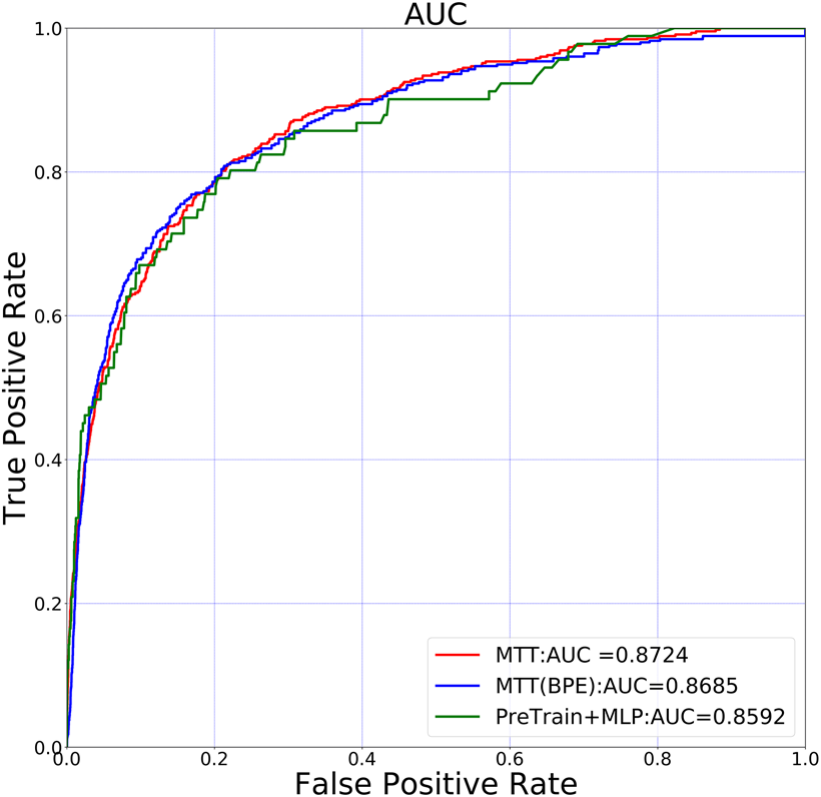
A prediction performance comparison of MTT with various encoders. The experimental results show that the performance of MTT-based predictor can be improved by adopting of multiple tokenization

The dimension of the feature vector was set at 128, the heads of attention at 4, the layer number of transformers at 5, and the learning rate at 0.001. Finally, C–T obtained an AUC of 0.8724. Figure 8 shows the comparisons of MTT with various encoders. MTT represents the classifier using MTT with ECFP1, ECFP2, and BPE tokenization. MTT(BPE) represents the classifier using MTT with only BPE tokenization. PreTrain + MLP represents the vectors derived by classification using Pretrain do not use the Transformer layer for presentation learning. Experimental results show that the adoption of multiple tokenization can improve prediction performance.

**Fig. 9:**
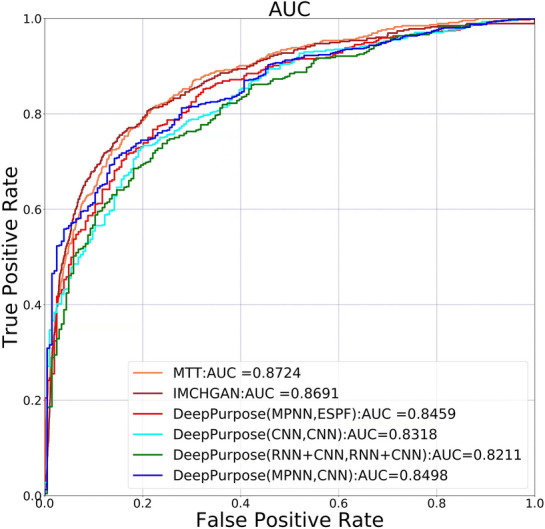
Prediction performance comparison of our proposed method with other predictors. The MTT-based prediction model was compared with two recently proposed compound-target interaction models in our released natural product compound-target interaction dataset. DeepPurpose(CNN, CNN) represents the prediction model in [24] with a Convolutional neural network as feature encoders both for compounds and proteins. Other DeepPurpose symbols were interpreted similarly. Ten-fold cross-validation was used to evaluate the prediction performance of the various compared C–T models

In order to make the compound-target interaction prediction ability of MTT-based prediction tool proposed in this paper more convincing, we made experimental comparison between MTT and other two recent compound-target interaction prediction methods [24, 25]. The comparison results are shown as follows (Fig. 9). The experimental results show MTT-based prediction model is superior to the compared methods in our released natural product compound-target interaction dataset.

### Website interface

**Fig. 10:**
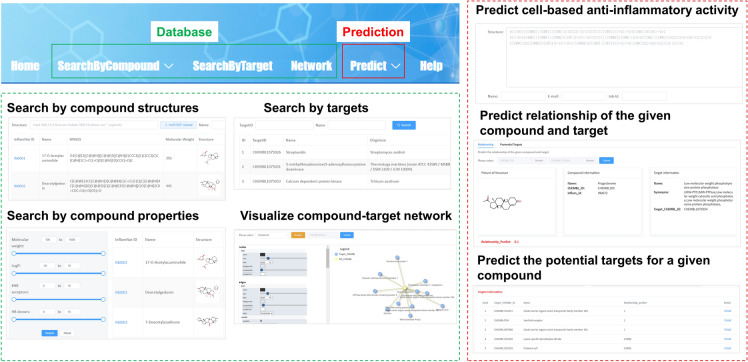
An overview of the InflamNat website. The database domain of the website allows users to search for compound structure and properties, anti-inflammatory cellular activity, and molecular targets, as well as visualize the compound-target network. The prediction domain provides tools to predict the inhibitory activity of NO production and the compound-target relationship

InflamNat (http://www.inflamnat.com/ or http://39.104.56.4/) combined one database and two machine learning-based predictive tools (Fig. 10). Users can search the database using several approaches: (1) providing the NP structure (SMILES, MOL2, SDF), (2) selecting a range of molecular properties, and (3) entering the name or ChEMBL ID of target proteins. The retrievable data included the basic compound information (Name, IUPAC, SMILES, InChiKey, ChEMBL_ID, PubChem_ID, compound class, and origin organism), physicochemical properties (MW, molecular formula, LogP, #HBA, #HBD, and #RotB), cell-based anti-inflammatory bioactivity (inhibiting the production of NO, PGE2, IL-1, and cytotoxicity), and protein targets (IC_50_ < 50 μM). The NP-target network can be visualized by downloading the complete dataset (including negative NP-target interaction data) via the links on the home page. The database will be updated annually to expand the number of anti-inflammatory compounds.

Furthermore, users can predict the anti-inflammatory activity of natural products by uploading their structures. The results will be sent via e-mail and presented as the probability of having an IC_50_ (inhibition of NO production in macrophages) < 50 μM. For InflamNat compounds and targets that are collected in the database but lack existing relationship data, one can predict the relationship of the given compound and target, as well as retrieve all the potential targets for a specific compound.

## Conclusion 

Machine learning is a valuable tool for drug development. However, the application of ML in the discovery of bioactive NPs has been limited by the lack of well-curated databases and user-friendly tools for chemists and pharmacologists. InflamNat aimed to support the discovery of NP-inspired anti-inflammatory drug leads via informatics approaches, including database and online predictive tools. This platform integrated the knowledge of physicochemical properties, cellular anti-inflammatory bioactivities, and molecular targets. This study was expected to promote the development of easily accessible informatics sources for NP-derived drug therapy in the treatment of other diseases, such as neurological diseases and cardiovascular diseases.

Nevertheless, the InflamNat platform still requires further improvements based on more readily available and robust experimental data. NO production is only associated with specific inflammation cell pathways, such as NF-κB, whereas different inflammatory diseases may involve other signaling pathways that are not characterized by NO levels but by other chemokines and cytokines. Therefore, predictive models based on pro-inflammatory factors other than NO should be studied in the future to cover a wide range of inflammatory conditions. It remains a challenge due to the lack of high-quality and adequate amount of data, especially for NPs. In this case, techniques, such as transfer learning, would be sufficient for treating limited datasets.

## Supplementary Information

**Additional file 1.** Introduction and user guide of the InflamNat website was also provided as supporting information.

**Additional file 2.** Physicochemical properties and cell-based anti-inflammatory activity of InflamNat Compounds.

**Additional file 3.** Molecular Targets of InflamNat Compounds.

**Additional file 4.** Basic information of the targets.

## Data Availability

The implemented code and experimental dataset are available online at http://www.inflamnat.com/ or http://39.104.56.4/ The source code of MTT-based prediction tools was uploaded to https://github.com/ljatynu/MTT.
