# Supplementary material for: InflamNat: web-based database and predictor of anti-inflammatory natural products
Source: J Cheminform. 2022 Jun 4;14:30. doi: 10.1186/s13321-022-00608-5 (PMC9167499; doi:10.1186/s13321-022-00608-5)

**Introduction to InflamNat**

InflamNat is a comprehensive and FREE online platform to assist the development of natural anti-inflammatory agents. InflamNat integrated **one** **database** and **two** **predictive tools**. With this website, you can:

- **Search by Compound:** search for the anti-inflammatory activity and molecular targets of any natural product. Similarity search is supported.
- **Search by Target:** search for related natural products based on a certain molecular target
- **Predict the anti-inflammation activity** of natural products
- **Predict the compound-target relationship** for the compounds and targets collected in the database but without existed relationship data
- **Download the complete database** containing the compounds and bioactivity information.

InflamNat database collected more than **1351** (*up to 2021*) natural compounds tested in anti-inflammatory assays, providing their physicochemical properties, cell-based anti-inflammatory bioactivities and molecular targets (if identified). The core data of InflamNat database was the cell-based anti-inflammation bioactivity manually curated from literature, such as the inhibition of nitric oxide (NO) or PGE2 production, and IL-1/6/8 secretion etc. The molecular targets of InflamNat compounds were collected from ChEMBL, with manually processing to identify the “real” targets that **directly influenced** by the compounds.

InflamNat also provides two machine learning-based predictive tool specifically designed for natural products:

One is to **evaluate the anti-inflammation potential** of a given natural product. The anti-inflammation activity was represented by **inhibition of NO production**, which is the classic inflammatory reaction and most commonly used indicator of inflammation, so that we can collect enough data for model construction. The structure and property of natural products are different from the synthesized compounds in many aspects. Therefore, this model was trained by natural products for the consistency of applicability domain.

The other is to **predict the relationship between the compounds and targets** collected in the database, but without experimentally verified data. Since the molecular targets of a large number of anti-inflammatory natural products have not been identified, this tool is useful for in-depth study and repurposing of these compounds.

**1. Search by Compound**

You can query the database by providing molecule structures or specifying the properties (molecular weight, logP, number of hydrogen bond acceptors or donors).

- **
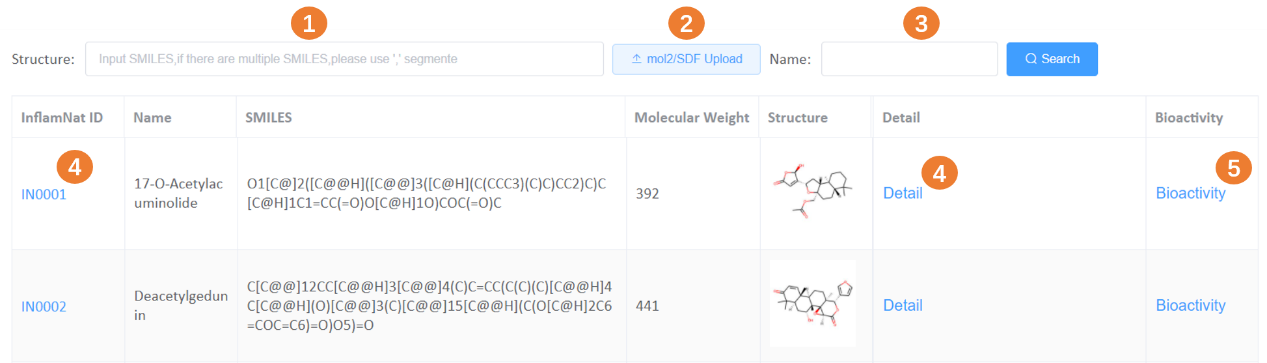
Search by compound structures**

**1.** Input SMILES format of the natural product (NP) structure, use ‘,’ to separate multiple SMILES.

**2.** Upload MOL2 or SDF format of NP structures.

**3.** Input the name of NP.

**4.** Access the detail of the NP through the links.

**5.** Access the anti-inflammatory activity and molecular targets data through this link.

- **Search by compound properties**


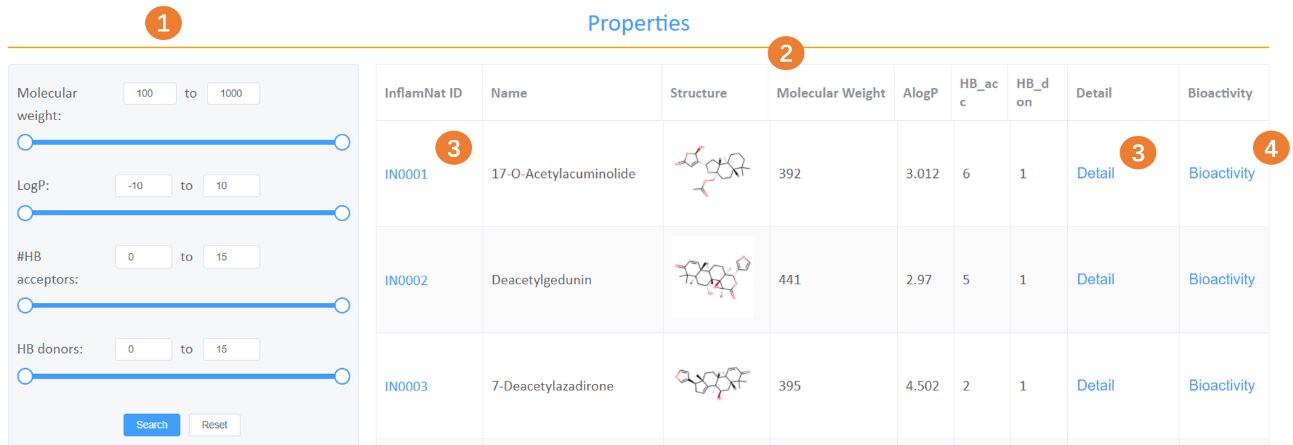


**1.** Define the range of the properties

**2.** List of the InflamNat NPs that fit into the property requirements

**3.** Access the detail of the NP through the links.

**4.** Access the anti-inflammatory activity and molecular targets data through this link.


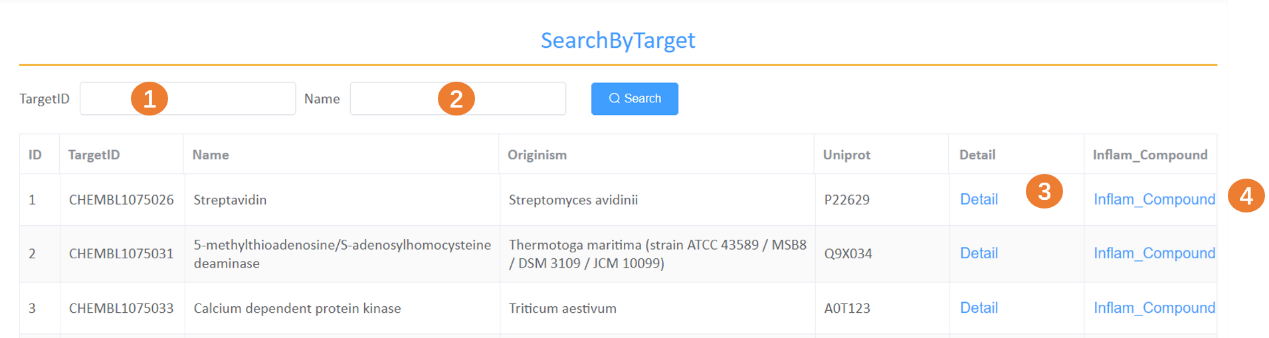
**2. Search by target**

**1.** Input the ChEMBL ID of the protein

**2.** Input the name of the protein

**3.** Access the detail of the protein through the links.

**4.** Access the InflamNat NPs that target this protein


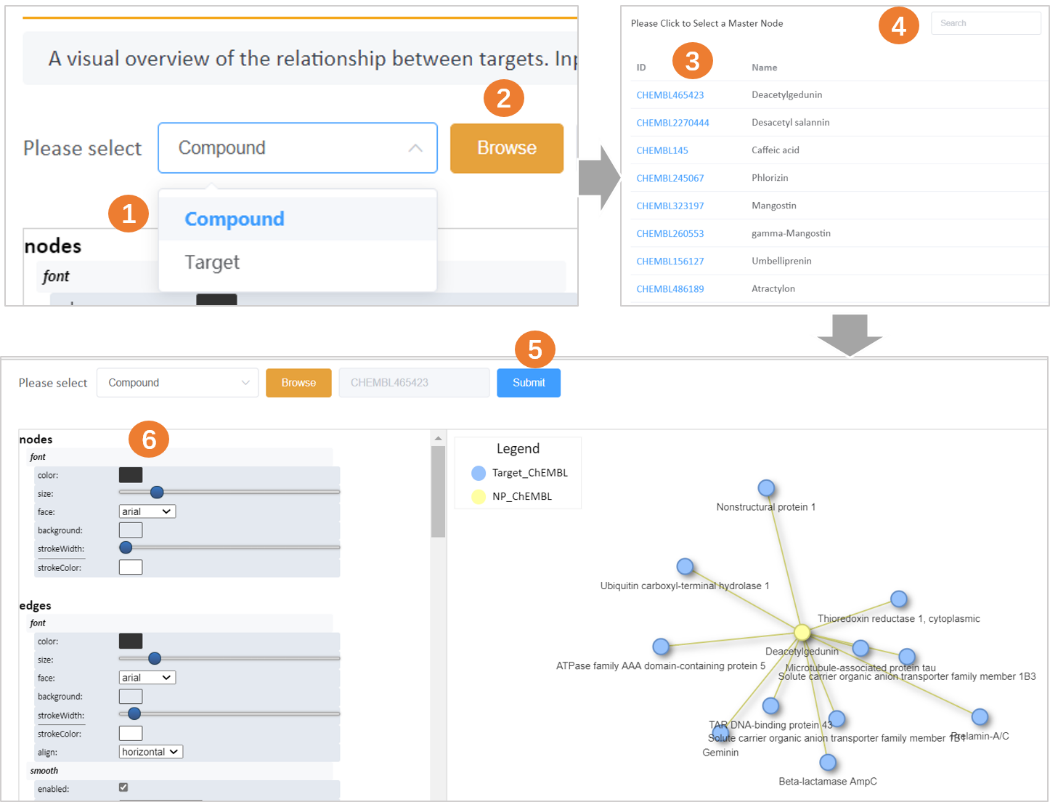
**3. Visualize the compound-target network**

**1.** Select *Compound* or *Target* as the central node

**2.** Click to browse the list of compounds or targets

**3.** Click to select this compound or target

**4.** Search by entering ChEMBL ID or Name

**5.** Click to visualize the network (the targets that directly influenced by the givin compound, or the compounds that directly influence the given target)

**6.** Adjust the visualization parameters


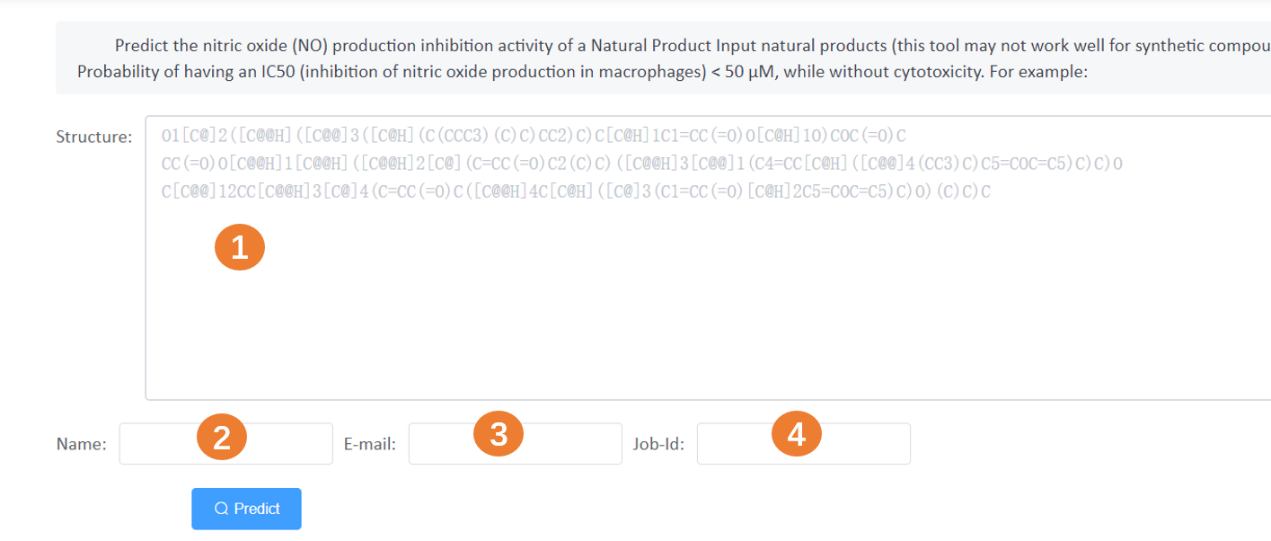
**4. Predict the anti-inflammatory activity (NO production inhibition) of given NPs**

**1.** Enter the SMILES of your query NPs

**2.** Enter a name that you want to be called

**3.** Provide a valid email address to receive the predict results, check the *Trash* of the mailbox if necessary.

**4.** Enter a ID for your job

**5.** Click to start the prediction

**5. Predict the compound-target relationship**

- **Predict the relationship of the given compound and target**


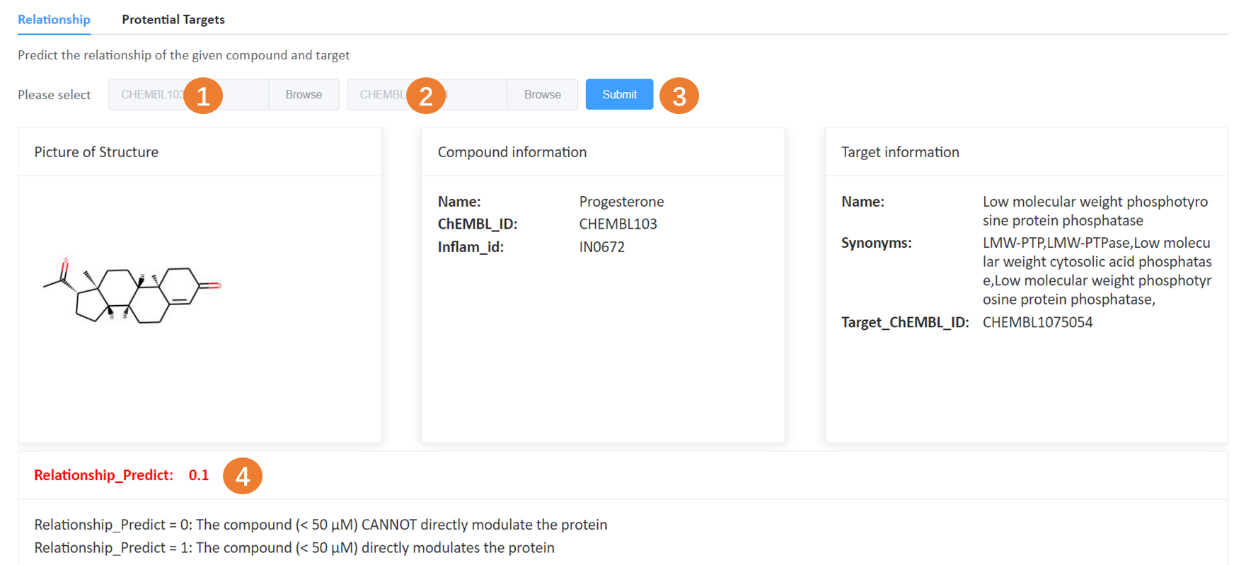


**1.** Browse to select a compound

**2.** Browse to select a target

**3.** Submit to get the prediction result

**4.** Relationship_Predict = 0: The compound (< 50 μM) CANNOT directly modulate the protein.

Relationship_Predict = 1: The compound (< 50 μM) directly modulates the protein.

- **Predict the potential targets for the given compound(with the predicted score >0.9)**


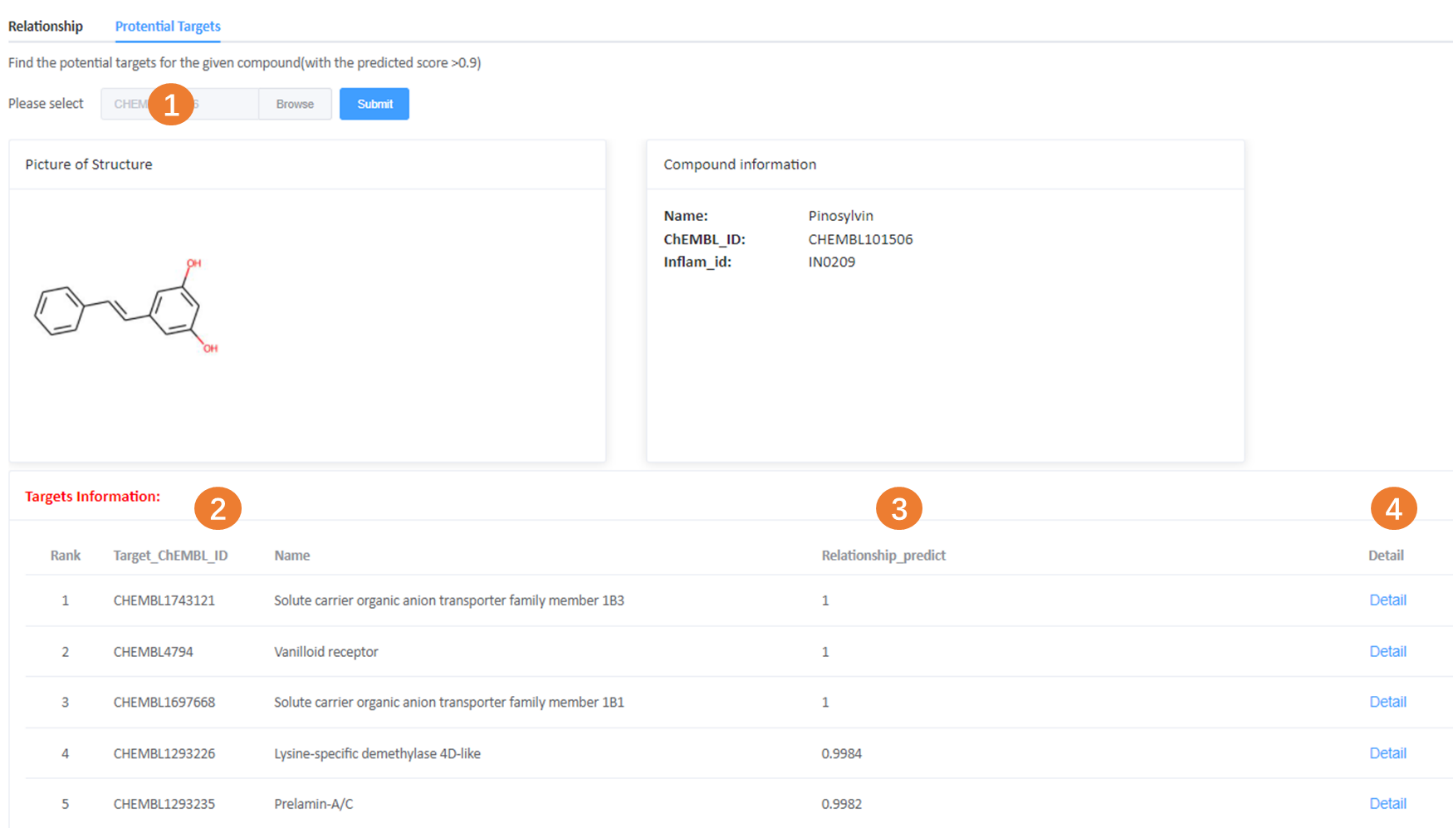


**1.** Browse to select a compound

**2.** Predicted targets for the compound with the score > 0.9

**3.** Predict score

**4.** Click to see the detail of the target

**6. Download the complete data**


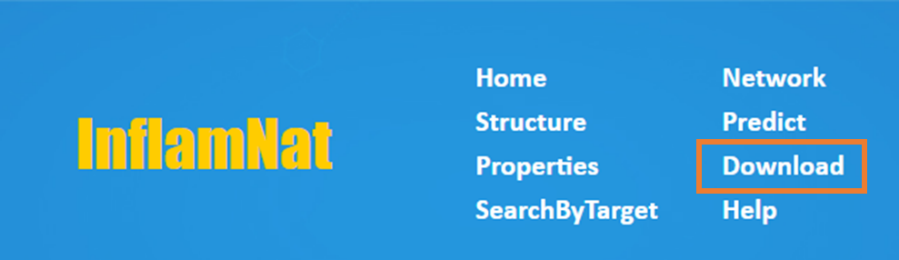


**1. Search by Compound**

You can query the database by providing molecule structures or specifying the properties (molecular weight, logP, number of hydrogen bond acceptors or donors).

- **
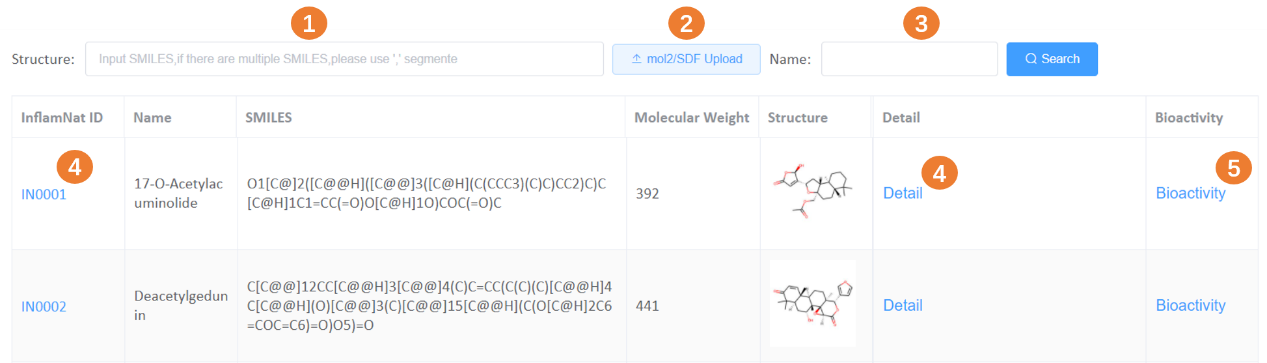
Search by compound structures**

**1.** Input SMILES format of the natural product (NP) structure, use ‘,’ to separate multiple SMILES.

**2.** Upload MOL2 or SDF format of NP structures.

**3.** Input the name of NP.

**4.** Access the detail of the NP through the links.

**5.** Access the anti-inflammatory activity and molecular targets data through this link.

- **Search by compound properties**


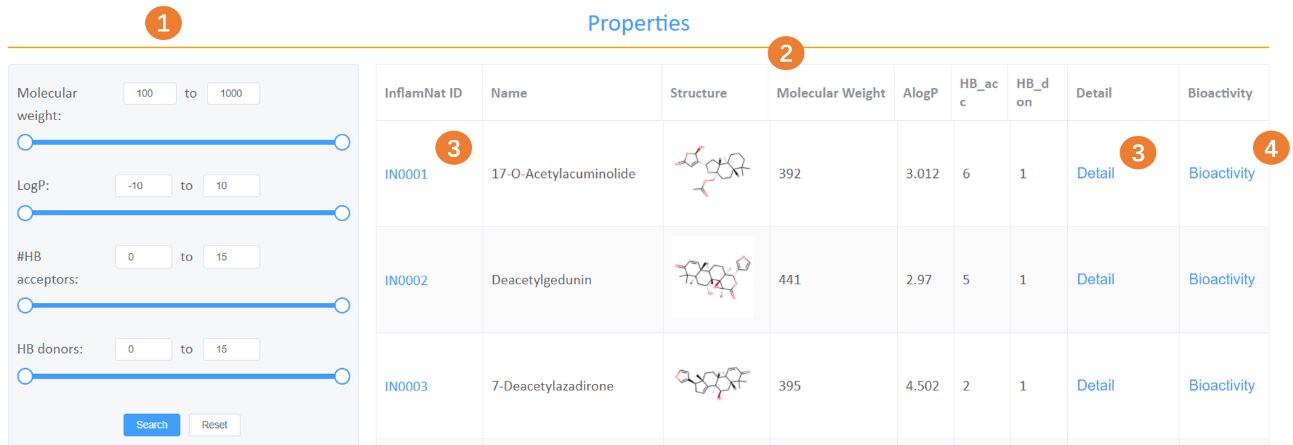


**1.** Define the range of the properties

**2.** List of the InflamNat NPs that fit into the property requirements

**3.** Access the detail of the NP through the links.

**4.** Access the anti-inflammatory activity and molecular targets data through this link.

- **Search by target**


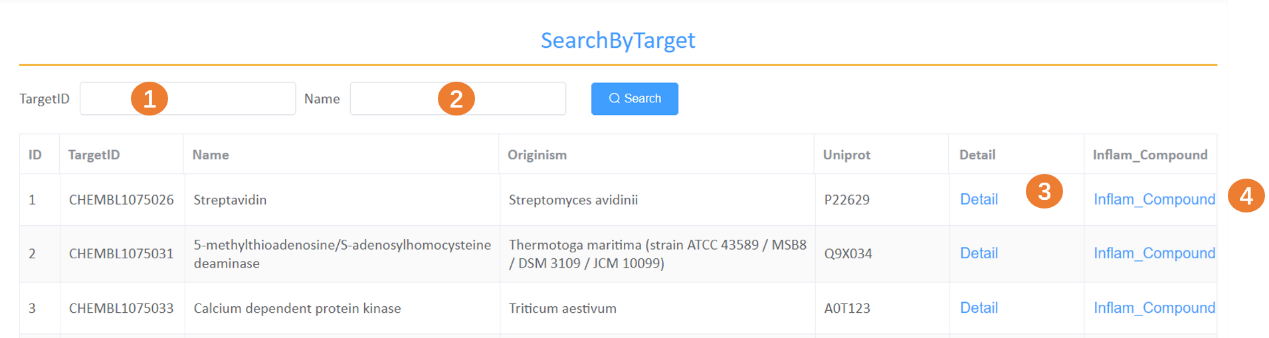


**1.** Input the ChEMBL ID of the protein

**2.** Input the name of the protein

**3.** Access the detail of the protein through the links.

**4.** Access the InflamNat NPs that target this protein

-
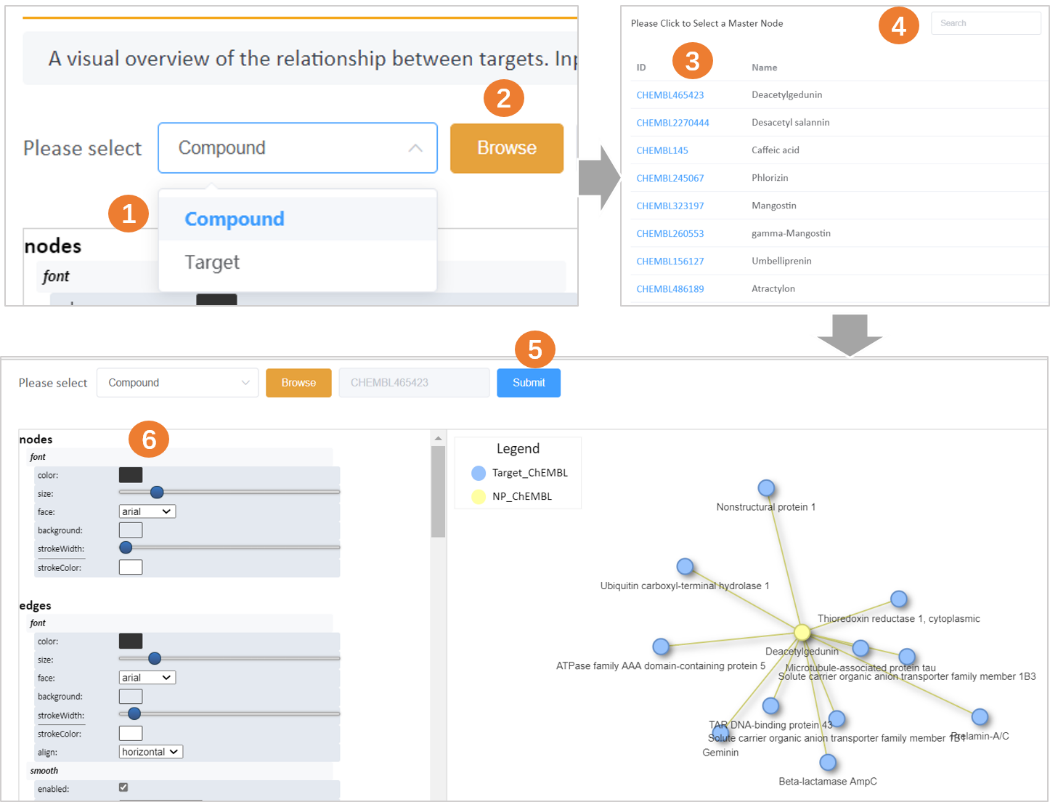
**Visualize the compound-target network**

**1.** Select *Compound* or *Target* as the central node

**2.** Click to browse the list of compounds or targets

**3.** Click to select this compound or target

**4.** Search by entering ChEMBL ID or Name

**5.** Click to visualize the network (the targets that directly influenced by the givin compound, or the compounds that directly influence the given target)

**6.** Adjust the visualization parameters

-
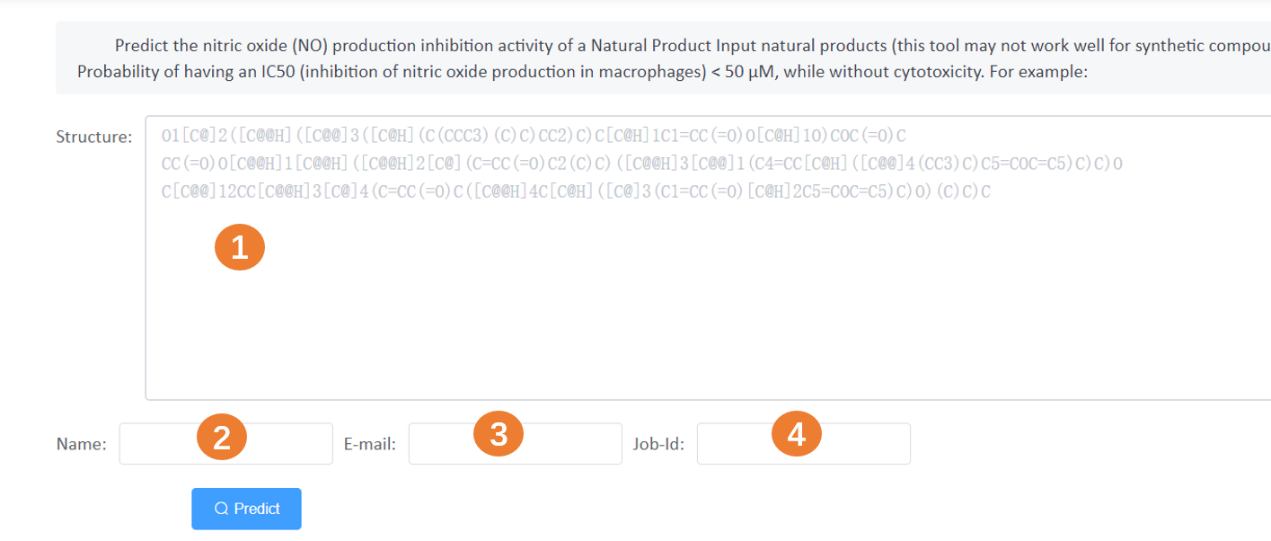
**Predict the anti-inflammatory activity (NO production inhibition) of given NPs**

**1.** Enter the SMILES of your query NPs

**2.** Enter a name that you want to be called

**3.** Provide a valid email address to receive the predict results, check the *Trash* of the mailbox if necessary.

**4.** Enter a ID for your job

**5.** Click to start the prediction

- **Predict the compound-target relationship**
- **Download the complete data**


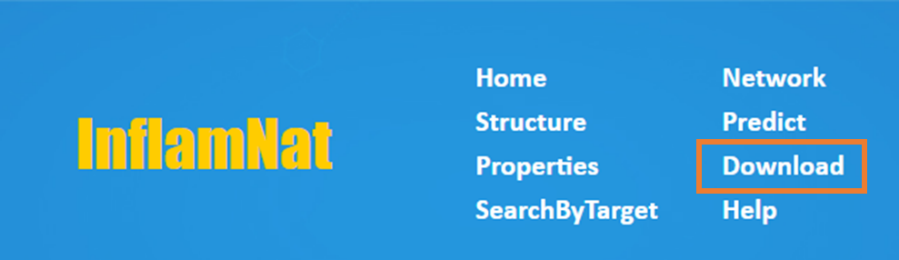

Supplement: Supplementary file 1 — Additional file 1. Introduction and user guide of the InflamNat website was also provided as supporting information. [file 13321_2022_608_MOESM1_ESM.docx]
